# Epithelial Mutant p53 Promotes Resistance to Anti-PD-1-Mediated Oral Cancer Immunoprevention in Carcinogen-Induced Mouse Models

**DOI:** 10.3390/cancers13061471

**Published:** 2021-03-23

**Authors:** Jin Wang, Yuan Hu, Vicente Escamilla-Rivera, Cassandra L. Gonzalez, Lin Tang, Bingbing Wang, Adel K. El-Naggar, Jeffrey N. Myers, Carlos Caulin

**Affiliations:** 1 Department of Head and Neck Surgery, The University of Texas MD Anderson Cancer Center, Houston, TX 77030, USA; wangj10@sj-hospital.org (J.W.); huyuan_ent@hust.edu.cn (Y.H.); cassandralg@icloud.com (C.L.G.); ltang@arizona.edu (L.T.); bingwang@mdanderson.org (B.W.); jmyers@mdanderson.org (J.N.M.); 2 Department of E.N.T., Shengjing Hospital of China Medical University, Shenyang 110004, China; 3 Department of Otorhinolaryngology, Union Hospital, Tongji Medical College, Huazhong University of Science and Technology, Wuhan 430022, China; 4 Department of Otolaryngology—Head & Neck Surgery, University of Arizona, Tucson, AZ 85724, USA; vicentee@arizona.edu; 5 Department of Medicine, University of Arizona, Tucson, AZ 85724, USA; 6 Department of Pathology, The University of Texas MD Anderson Cancer Center, Houston, TX 77030, USA; anaggar@mdanderson.org; 7 The University of Arizona Cancer Center, University of Arizona, Tucson, AZ 85724, USA

**Keywords:** immunoprevention, oral cancer, mutant p53, PD-1, premalignancy

## Abstract

**Simple Summary:**

Immune checkpoint blockade with anti-PD-1 antibodies blocks the development of oral squamous cell carcinomas (OSCCs) in preclinical models. Understanding whether the genetic alterations that accumulate during oral cancer development affect the response to PD-1 inhibitors is critical to identify patients who may benefit from immunoprevention interventions. Using genetically engineered mouse models that develop carcinogen-induced oral tumors that differ on the mutational status of the *p53* gene, we demonstrated that expression of gain-of-function mutant *p53* in the epithelial cells of the oral lesions promotes resistance to the immunopreventive effects of anti-PD-1. These novel findings could guide patient-specific strategies for oral cancer immunoprevention based on *p53* profiling.

**Abstract:**

Oral squamous cell carcinoma (OSCC) develops through the multistep malignant progression of squamous epithelium. This process can be prevented by PD-1 blockade in a mouse model for oral carcinogenesis. OSCCs exhibit a high incidence of *p53* mutations that confer oncogenic gain-of-function (GOF) activities that promote resistance to standard therapies and poor clinical outcomes. To determine whether epithelial *p53* mutations modulate anti-PD-1-mediated oral cancer immunoprevention, we generated mouse models for oral carcinogenesis by exposing mice carrying epithelial-specific *p53* mutations to the carcinogen 4NQO. Consistent with the oncogenic functions of mutant *p53*, mice with OSCCs expressing the *p53^R172H^* GOF mutation developed higher metastasis rates than mice with loss-of-function (LOF) *p53* deletion or with wild-type *p53*. Throughout oral cancer progression, pre-invasive and invasive lesions showed a gradual increase in T-cell infiltration, recruitment of immunosuppressive regulatory T-cells (Tregs), and induction of PD-1/PD-L1 immune checkpoint proteins. Notably, while PD-1 blockade prevented the development of OSCCs in mice with wild-type *p53* or *p53* deletion, GOF *p53^R172H^* abrogated the immunopreventive effects of anti-PD-1, associated with upregulation of IL17 signaling and depletion of exhausted CD8 cells in the microenvironment of the *p53^R172H^* tumors. These findings sustain a potential role for *p53* profiling in personalized oral cancer immunoprevention.

## 1. Introduction

Oral cancer is the most frequent malignancy of the head and neck and the sixth most common human cancer worldwide, with five-year survival rates that remain at 50% [1,2]. Over 90% of the oral cancers are squamous cell carcinomas (OSCCs) that develop from the oral squamous epithelium following a multistep process of malignant progression that involves the acquisition of genetic and phenotypic alterations promoted by tobacco and/or alcohol as the main etiological factors [3,4]. Frequently, OSCC patients develop secondary independent tumors in the adjacent mucosa of pre-existing OSCCs, a process known as “field cancerization” that suggests the presence of multiple oral premalignant lesions (OPLs) in these patients [5]. Specific molecular markers that predict high risk of progression of OPLs are currently lacking, although loss-of-heterozygosity (LOH) in certain chromosomal regions such as 9p21 and 3p14 has been shown to be associated with shorter time of progression to OSCC [6].

To overcome tumor-promoting effects of carcinogens, immune surveillance mechanisms guard against oral cancer development, as evidenced by the higher risk of oral cancer development in immunosuppressed transplant recipients compared to the general population [7,8]. However, during tumor development, the immunoprotective function of the immune system may be compromised, at least in part, by the activation of immune checkpoints that block anti-tumor immunity [9,10]. Indeed, reactivation of the immune system using immune checkpoint inhibitors has been shown to be a powerful strategy in cancer therapy. Recent clinical trials with blocking antibodies for the immune checkpoint molecule programmed cell death protein 1 (PD-1) have shown to improve survival in patients with advanced head and neck squamous cell carcinoma [11,12,13]. Moreover, our previous studies showed that PD-1 blockade can prevent the malignant progression of OPLs, in a mouse model for oral carcinogenesis, supporting the preventive potential of immune checkpoint inhibition to contain oral cancer development [14].

Immune checkpoint inhibitors are increasingly used in the management of oral cancer patients, but the response rate is approximately 20% and the factors underlying this differential response have not been identified [11,12,13]. Progressive acquisition of genetic alterations in epithelial cells during oral cancer development plays a major role in the response of the tumors to standard therapies, including chemotherapy and radiation [15]. However, it is presently unclear how cancer-specific mutations affect the response to immune checkpoint inhibitors.

The *TP53* gene (also known as *p53*) is the most frequently mutated gene in OSCCs [16,17,18]. Most cancer-associated *p53* mutations are missense mutations that encode mutant forms of *p53*, some of which, in addition to losing wild type p53 functions, acquire oncogenic gain-of-function (GOF) activities [19,20]. High-risk *p53* mutations have been found to be associated with poor survival and lack of response to chemotherapy in patients with head and neck cancer [21,22,23,24]. Mutations in *p53* have also been found in up to 30% of the OPLs, suggesting that these mutations arise early during oral tumor development and may impact the progression of OPLs and their response to preventive strategies [25,26].

In this study, we assessed whether *p53* mutations affect the potential of anti-PD-1 antibodies to prevent the malignant progression of OPLs. To conduct preclinical studies for immunoprevention in the context of *p53* mutations, we generated genetically engineered mice in which the endogenous *p53* GOF mutation *p53^R172H^* (equivalent to human *p53^R175H^*), or a loss-of-function (LOF) *p53* deletion, were activated in the oral epithelium. These mice were exposed to the tobacco-surrogate 4-Nitroquinoline 1-oxide (4NQO), a water-soluble carcinogen that induces OPLs that may progress to advanced carcinomas following stepwise changes that resemble the gradual accumulation of histological and molecular abnormalities observed during human oral cancer progression [27,28,29,30,31]. The mice were treated with anti-PD-1 antibodies to determine whether *p53* mutations modulate the immunopreventive effects of immune checkpoint inhibitors.

## 2. Materials and Methods

### 2.1. Mouse Models

Mice for conditional activation of the *p53^R172H^*mutation (Neo-*Trp53^R172H^* mice) and *p53* deletion (floxed-*p53*), K5.CrePR1 transgenic mice, and genotyping protocols have been previously described [32,33,34]. The *p53*-GOF mutation *p53^R172H^* or a *p53*-LOF mutation (homozygous *p53* deletion) were activated in oral epithelial cells by crossing Neo-*Trp53^R172H^* and floxed-*p53* mice with K5.CrePR1 mice that drive activation of conditional alleles to stratified epithelia [35]. The following groups of mice were generated: (1) Mice with epithelial activation of *p53^R172H^* and deletion of the remaining *p53* allele (*p53*-GOF); (2) mice with homozygous deletion of *p53* (*p53*-LOF); (3) and wild type *p53* mice (*p53*-WT). Experimental mice were generated in a mixed strain background (C57BL/6;129Sv) and all comparative studies were conducted using littermates. The genetic background of the mouse experimental groups was confirmed using the Genetic Monitoring system from Transnetyx (Cordova, TN, USA) with a panel of 120 strain-specific single nucleotide polymorphisms (SNPs) across the mouse genome (Figure S1). For tumor studies, mice were exposed to 4NQO (100 μg/mL) in the drinking water for 8 weeks. All animals showing excessive tumor burden as per Institutional Animal Care and Use Committee (IACUC) guidelines were euthanized, and tumors were harvested for histopathologic and molecular analyses. The neck lymphoid nodes and lungs were harvested for metastasis analysis.

All experimental protocols using mice followed the ARRIVE guidelines (Animal Research: Reporting of In Vivo Experiments) and were approved by Institutional Animal Care and Use Committees (IACUC) of The University of Texas MD Anderson Cancer Center (#00000950-RN01) and The University of Arizona (#18-402).

### 2.2. Immunoprevention Preclinical Studies

The following experimental groups were studied: *p53*-WT[IgG] (*n* = 21); *p53*-WT[anti-PD-1] (*n* = 20); *p53*-LOF[IgG] (*n* = 19); *p53*-LOF[anti-PD-1] (*n* = 18); *p53*-GOF[IgG] (*n* = 24); *p53*-GOF[anti-PD-1] (*n* = 19). Oral lesions were induced in 6-8-week-old mice by 4NQO (100 μg/mL) administration to the drinking water, for 8 weeks. Four weeks after completion of the 4NQO treatment the mice were injected intraperitoneally with 250 μg/mouse of anti-PD-1 (RMP1-14) or IgG2a (250 μg/mouse), both from Bio×Cell (West Lebanon, NH, USA), twice a week for 4 weeks. Mice were euthanized for tissue retrieval six weeks after completion of the antibody injection.

### 2.3. Histology and Immunohistochemistry Analyses

Tissue and tumors were fixed in 10% neutral-buffered formalin at room temperature overnight, then transferred to 75% ethanol and embedded in paraffin. Histologic sections (5µm) were stained with hematoxylin and eosin or processed for immunohistochemical analysis (IHC). Dysplastic cellular changes limited to the lower and middle thirds of squamous epithelium were classified as mild or moderate dysplasia, respectively, and considered low-grade lesions. Lesions in which these changes involved over two-thirds of the epithelium were graded as severe dysplasia. Invasive carcinoma was defined as a lesion with invasion through the basement membrane into the subepithelial tissues. Severe dysplasia and carcinoma were considered high-grade lesions [14,36]. IHC was performed using the Leica Bond Max automated stainer (Leica Biosystems, Buffalo Grove, IL, USA) with the following primary antibodies: Anti-CD3 antibody (ab5690) from Abcam (Cambridge, MA, USA); anti-PD-1 (#135202) and anti-K14 (#905301) from BioLegend (San Diego, CA, USA); antibodies for CD4 (#14-0042-85) and Foxp3 (#14-5773) from eBioscience (Santa Clara, CA, USA); anti-CD8a (#361003) from Synaptic Systems (Göttingen, Germany); anti-PD-L1 (#17952-1-AP) from Proteintech Group (Chicago, IL, USA). Images were captured on a DMLA microscope equipped with a DFC310 FX camera (Leica Microsystems, Buffalo Grove, IL, USA). Staining for CD3, CD4, CD8, FOXP3, PD-1, and PD-L1, was scored as density of cells, defined as the number of positive cells per mm^2^. Mice that did not receive 4NQO, lacking oral lesions, were used as control. The number of samples analyzed in each experiment is listed in Table S1.

### 2.4. RNA and DNA Purification

Oral lesions were macro-dissected from 10µm histological sections using a H&E replica slide as a reference. The sections were de-paraffinized with 100% xylene, centrifuged, washed with 100% Ethanol to completely remove xylene, and dried at room temperature. RNA or DNA were purified form 10–20 sections from each sample, using the High Pure FFPET RNA kit (Roche, Basel, Switzerland) and the ReliaPrep™ FFPE gDNA Miniprep System (Promega, Madison, WI, USA), respectively, according to manufacturer guidelines.

### 2.5. Nanostring Analysis

RNA samples were quantitated using the Qubit 3 Fluorometer (Thermo Scientific, Waltham, MA, USA), evaluated for purity using the NanoDrop One Spectrophotometer (Thermo Scientific) and assessed for sample integrity with the 2100 Bioanalyzer (Agilent Genomics). Target-specific, fluorescence-labeled CodeSet and Reporter probes were hybridized to 300 ng RNA for 20 h using the nCounter PanCancer Mouse Immune Profiling panel (nanoString). The hybridized products were added to the robotic nCounter Prep Station for automated sample processing for the remaining steps. The cartridge was then moved to the nCounter Digital Analyzer MAX (NanoString) where the samples were scanned, and fluorescent targets counted. The raw counts (Table S2) were compiled with nSolver software (NanoString) and used for downstream analysis. Scores for 14 different immune cell types were generated with nSolver software, using default settings. Differential gene expression and hierarchical clustering was conducted using the DESeq package in Bioconductor [37]. Differentially expressed genes were ranked according to their Wald statistic value, and the ranked gene lists were used for Gene Set Enrichment Analysis (GSEA) analysis [38].

### 2.6. p53 Sequencing

DNA was amplified with DreamTaq DNA Polymerase (Thermo Scientific, Waltham, MA, USA), using specific primers for exons 2-11 and their flanking intron sequences (Table S3). For RNA sequencing, First Strand cDNA was generated with RevertAid First Strand cDNA Synthesis Kit (Thermo Scientific) and amplified with DreamTaq DNA Polymerase. The PCR products were purified with DNA Clean & Concentrator columns (Zymo Research, Irvine, CA, USA) and Sanger-sequenced with forward and reverse primers at the University of Arizona Genetics Core. Twelve p53-WT tumors (seven papillomas and five SCC), and eight p53-GOF tumors (four papillomas and four SCCs) were sequenced. 

### 2.7. Statistical Analyses

Tumor development and mouse survival was represented using Kaplan–Meier plots and the differences between groups were assessed by the log-rank test. Mouse strain background, immunohistochemistry counts, gene expression scores, and number of oral lesions in the different groups were compared using the 2-tailed Student *t*-test. Boxplots were generated in R with the ggplot2 package. Difference in metastasis rates was assessed by the Chi-square method. *p*-values < 0.05 were considered statistically significant.

## 3. Results

### 3.1. Mutant p53^R172H^ Promotes Metastasis in 4NQO-Induced Oral Tumors 

To assess the distinctive roles of *p53* GOF and LOF mutations during oral cancer development, we generated mice in which the endogenous *p53*-GOF mutation *p53^R172H^* or a *p53*-LOF mutation (homozygous *p53* deletion) were activated in oral squamous epithelial cells [35]. To induce oral lesions that differ in their *p53* status, these mice were exposed to the carcinogen 4NQO in the drinking water and they were monitored weekly for the development of exophytic lesions in the tongue and oral mucosa.

**Figure 1:**
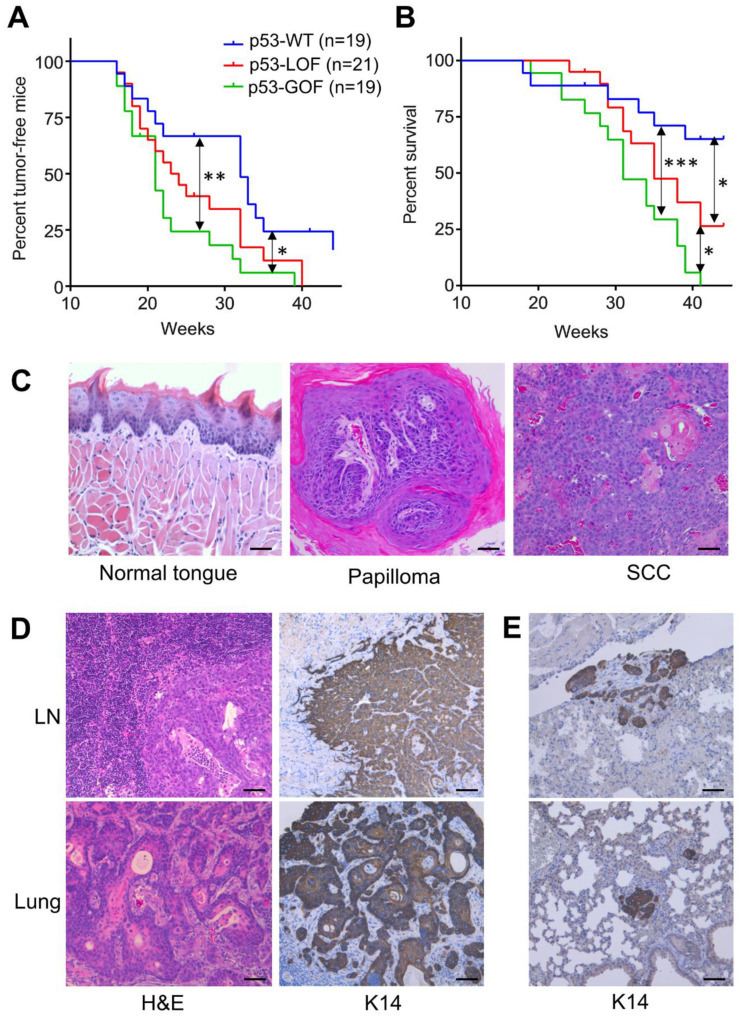
Mutant *p53^R172H^* promotes metastasis in oral tumors initiated by 4-Nitroquinoline 1-oxide (4NQO). (**A**) Kinetics of 4NQO-induced oral tumor development in mice with the indicated p53 genotypes. (**B**) Survival of mice with 4NQO-induced oral tumors that differ on their p53 status. (**C**) Representative images of the normal tongue epithelium, benign papillomas, and squamous cell carcinomas (SCCs) that developed in mice upon 4NQO treatment. Bar = 100 μm. (**D**) H&E staining of metastasis that developed in lymph nodes (LN) and lung (left panels), and their respective K14 staining (right panels). Bar = 100 μm. (**E**) K14 staining for two independent lung samples showing the presence of micrometastasis. Bar = 100 μm. * *p* < 0.05, ** *p* < 0.01, *** *p* < 0.001.

Mice with *p53* GOF or LOF mutations developed oral tumors significantly faster than *p53*-WT mice (Figure 1A), with a median appearance time of 21 weeks for *p53*-GOF mice, 23.5 weeks for *p53*-LOF mice and 32 weeks for *p53*-WT mice (Table S4). Although *p53*-GOF tumors developed slightly faster than *p53*-LOF tumors, this difference was not statistically significant, suggesting that both *p53* GOF and LOF mutations accelerate carcinogen-induced oral tumor development with similar potential. To determine the contribution of *p53* mutations to oral cancer metastasis, mice were monitored until development of morbidity endpoints according to IACUC regulations. At this point, the mice were euthanized, and tissue was harvested for histological evaluation. We found that *p53*-GOF mice reached morbidity endpoints significantly faster (31 weeks) than *p53*-LOF mice (35 weeks) and *p53*-WT mice, for which the median survival was considered to be >44 weeks because over 50% of the mice were still alive and healthy at the completion of the study, 44 weeks after initiation of the 4NQO treatment (Figure 1B and Table S4). Histological analysis of the oral exophytic lesions revealed that benign squamous papillomas and OSCCs developed in all genotypes (Figure 1C). Since the mice were maintained in the study until reaching the endpoints, we could not determine the rates of progression to SCC.

To investigate the role of *p53* GOF and LOF mutations in oral cancer metastasis, we harvested and processed neck lymph nodes and internal organs from these mice. Histopathological analysis of these samples identified squamous carcinoma metastasis in a subset of mice (Figure 1D). In addition, tissue sections were stained for Keratin 14 (K14) to detect the presence of micrometastases (Figure 1D,E). This analysis revealed that 30% of the *p53*-GOF mice had developed metastasis compared to only 5% in *p53*-LOF and *p53*-WT mice (Table S4). All metastases were found in the lungs, except for one instance to a lymph node. These findings indicate that the metastatic potential of the *p53*-GOF mutation *p53^R172H^* is much higher than that of *p53*-LOF in oral tumors induced by the carcinogen 4NQO. 

Since 4NQO has been reported to induce *p53* mutations that could potentially confound *p53*-based comparative analyses [30,31], we sequenced the *p53* gene in benign papillomas and SCCs. Of the 12 *p53*-WT tumors sequenced (seven papillomas and five SCCs), we identified only two *p53* mutations in SCCs, a synonymous AGC to AGT mutation (S310S), and an ACA to GCA mutation resulting in a T253A substitution (Figure S2A). The human equivalent to T253A (T256A) is a low-risk mutation that retains *p53* transcriptional activities, associated with similar favorable outcomes as *p53*-WT in OSCC [24,39]. No p53 mutations were found in *p53*-WT papillomas. Additionally, we confirmed the presence and expression of the R172H mutation in all *p53*-GOF tumors analyzed, and deletion of the remaining *p53*-floxed allele in these tumors (Figure S2B,C). Similarly, deletion of the *p53*-floxed allele was detected in all *p53*-LOF oral tumors analyzed (Figure S2D). Altogether, the data show no potential effects of 4NQO-induced *p53* mutations in our studies.

### 3.2. T-Cell Infiltration Increases Gradually during 4NQO-Induced Oral Cancer Progression 

**Figure 2:**
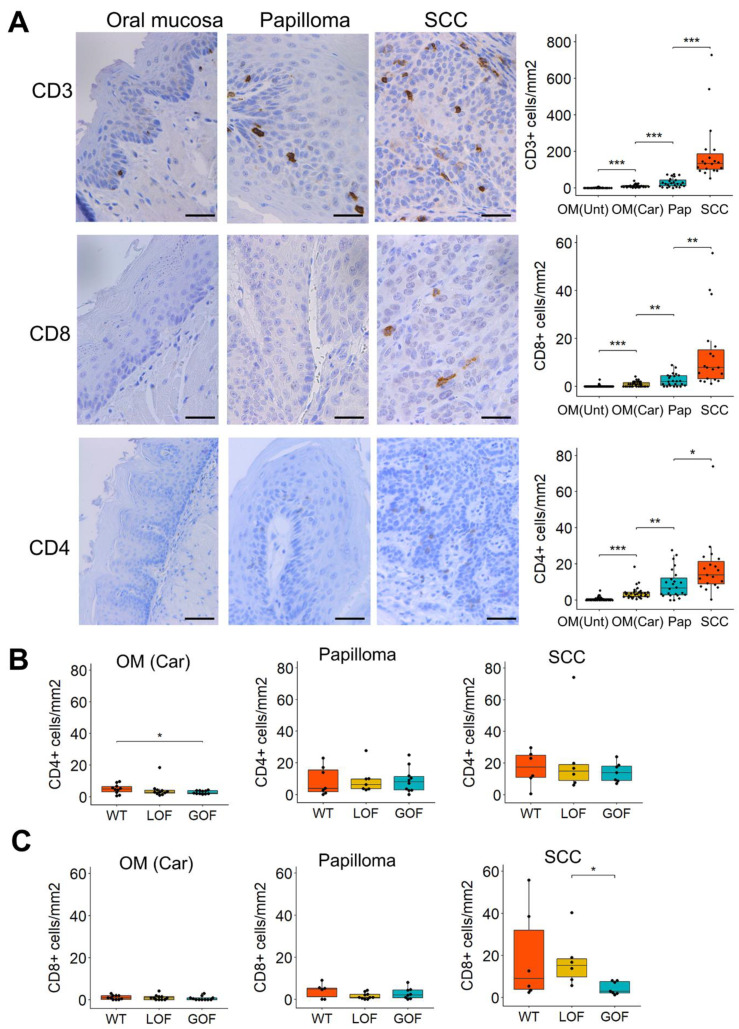
Gradual increase in T-cell infiltration during oral tumor progression. (**A**) Immunohistochemistry for CD3, CD4, CD8 in normal tongue and papillomas or SCCs induced by 4NQO. Quantification of the staining is shown on the right panels. Bar = 50 μm. (**B**) Analysis of the CD4 staining according to the p53 status of the mice. (**C**) Analysis of the CD8 staining according to the p53 status of the mice. OM(Unt): Oral mucosa from untreated mice; OM(Car): Oral mucosa from mice that had been treated with 4NQO; Pap: Papilloma; SCC: Squamous cell carcinoma. * *p* < 0.05, ** *p* < 0.01, *** *p* < 0.001.

The mouse models described above allow the evaluation of immune cell infiltrates in different stages of oral carcinogenesis and according to the *p53* status. When oral lesions were analyzed based on their histopathological features, irrespective of the *p53* status, we observed a gradual increase in the number of CD3+, CD8+, and CD4+ cells from the normal oral mucosa to benign papillomas and invasive SCCs (Figure 2A), indicating a progressive T-cell recruitment during oral tumor progression. Interestingly, the 4NQO-exposed oral mucosa that lacked detectable pathological lesions showed a significant increase in infiltrating T-cells compared to the oral mucosa from mice that did not receive 4NQO, as indicated by the higher number of CD3+, CD4+, and CD8+ infiltrating cells (Figure 2A). These observations indicate that 4NQO can induce immunogenic changes in the oral mucosa prior to the development of pathological lesions.

Comparative analysis of T-cell infiltration in the oral lesions according to their *p53* status revealed that the oral mucosa of *p53*-GOF mice that had been exposed to 4NQO contained lower levels of CD4+ cells than that of *p53*-WT tumors (Figure 2B). No significant changes in CD4+ T cell infiltration were noted in papillomas or SCCs that differ in their *p53* status (Figure 2B). CD8+ infiltration was significantly lower in *p53*-GOF SCCs than in *p53*-LOF SCC (Figure 2C), and lower than that of *p53*-WT SCCs, although this difference did not reach statistical significance, as high variability among samples was observed in the *p53*-WT tumors (Figure 2C). The CD8+ T-cell density in the oral mucosa of 4NQO-exposed mice and in papillomas was not affected by the *p53* status (Figure 2C). No significant differences associated to the *p53* status of the lesions were noted for CD3+ T-cell infiltration in premalignant or malignant lesions (Figure S3A). Together, these findings demonstrate a gradual increase in T-cell infiltration during 4NQO-induced oral cancer progression. The presence of the *p53* GOF mutation *p53^R172H^* in the epithelial cells of the oral lesions and tumors may affect the recruitment of the CD4+ and CD8+ cells during oral tumor progression.

### 3.3. Distinctive Immunosuppressive Profiles in Oral Lesions That Differ in Their p53 Status

The gradual accumulation of T-cells observed during oral cancer progression not only suggests increased immunogenicity, but also the potential activation of immunosuppressive mechanisms that may allow tumor progression. Activation of immune checkpoints and the presence of immunosuppressive regulatory T-cells (Tregs) in the tumor microenvironment are major mechanisms known to promote immune evasion in multiple malignancies, including head and neck cancers [9,11,12,40]. 

**Figure 3:**
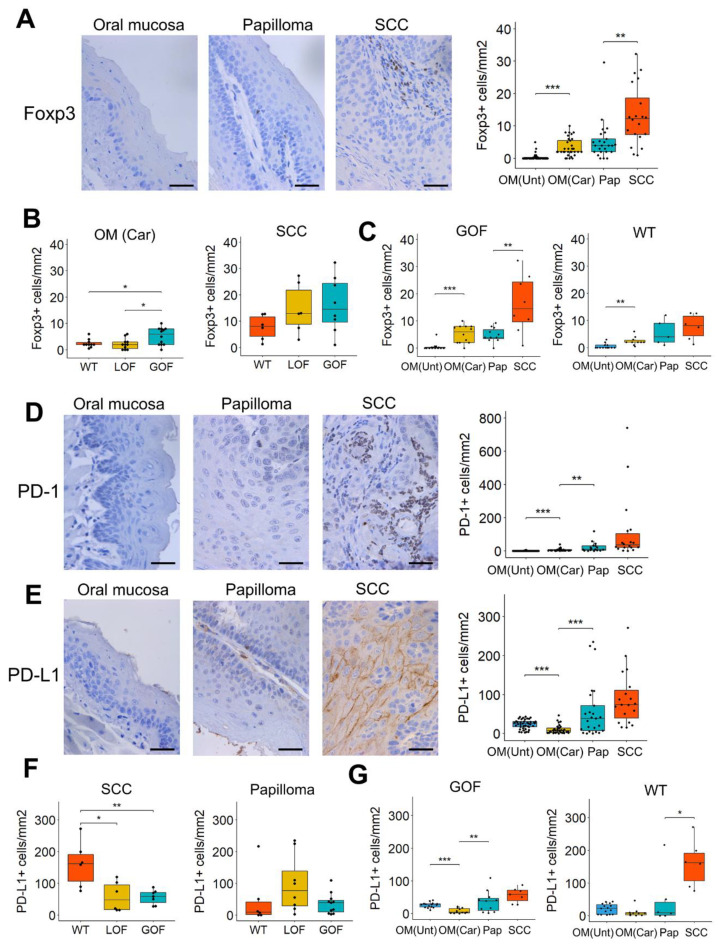
Immunosuppressive infiltrates in oral tumors induced by 4NQO. (**A**) Immunohistochemistry for Foxp3 in normal oral mucosa and papillomas or SCCs induced by 4NQO. Quantification of the staining is shown on the right panel. Bar = 50 μm. (**B**) Analysis of the Foxp3 staining according to the *p53* status of the oral lesions. (**C**) Analysis of the Foxp3 staining in *p53*-GOF mice (left panel) and *p53*-WT mice (right panel). (**D**) Immunohistochemistry for PD-1 in normal oral mucosa, papillomas, and SCCs induced by 4NQO. Quantification of the staining is shown on the right panel. Bar = 50 μm. (**E**) Immunohistochemistry for PD-L1 in normal oral mucosa and papillomas or SCCs induced by 4NQO. Quantification of the staining is shown on the right panel. Bar = 50 μm. (**F**) Analysis of the for PD-L1 staining according to the *p53* status of the oral lesions. (**G**) Analysis of the PD-L1 staining in *p53*-GOF mice (left panel) and p53-WT mice (right panel). OM(Unt): Oral mucosa from untreated mice; OM(Car): Oral mucosa from mice that had been treated with 4NQO; Pap: Papilloma; SCC: Squamous cell carcinoma. * *p* < 0.05, ** *p* < 0.01, *** *p* < 0.001.

To analyze Treg infiltration during 4NQO-induced oral carcinogenesis, we stained the oral lesions with an antibody for Foxp3, a transcription factor considered a master regulator of Tregs [41]. We found Foxp3+ infiltration to be higher in SCCs compared to papillomas and oral epithelium (Figure 3A), indicating increased Treg infiltration during malignant progression of oral tumors. Interestingly, oral mucosa of mice exposed to 4NQO showed similar counts of Foxp3+ cells as those observed in papillomas, suggesting an early recruitment of Tregs cells during oral carcinogenesis. Such early accumulation of Foxp3+ cells occurred preferentially in the carcinogen-exposed oral mucosa of *p53*-GOF mice, as evidenced by their higher levels of Foxp3+ infiltrates compared to those observed in *p53*-LOF and *p53*-WT mice (Figure 3B). No significant differences in Foxp3+ infiltration according to the *p53* status of the lesions were observed in papillomas and SCCs (Figure 3B and Figure S3B). Of note, Foxp3+ infiltration increased significantly (*p* < 0.05) from papillomas to SCC in *p53*-GOF mice, but not in *p53*-WT or *p53*-LOF mice (Figure 3C and Figure S3C). Overall, these data suggest that the appearance of early oral lesions and progression from benign papillomas to SCC may be promoted by the immunosuppressive functions of Tregs, preferentially in the presence of a *p53*-GOF mutation.

We also observed an increase in PD-1+ infiltrates and PD-L1 expression in epithelial cells of the oral lesions during oral cancer progression (Figure 3D,E). Although SCCs tend to express higher levels of PD-1/PD-L1 than papillomas, these differences were not statistically significant (*p* > 0.05). Notably, PD-L1 expression was lower in oral epithelial cells from mice that had been treated with 4NQO than in those from untreated mice (Figure 3E). This transient downregulation of PD-L1, together with the increased T-cell infiltration, may underlie the induction of a robust immune response that prevents cancer development in the carcinogen-exposed oral mucosa. We also found that the number of PD-L1+ cells was significantly lower in *p53*-GOF and *p53*-LOF SCCs compared to *p53*-WT SCCs (Figure 3F and Figure S3D), which may explain the lack of a robust increase in PD-L1 expression in SCCs compared to papillomas noted above (Figure 3D,E). Accordingly, PD-L1 increased significantly from papilloma to SCC only in *p53*-WT mice, but not in *p53*-GOF or *p53*-LOF mice (Figure 3G and Figure S3E). The *p53* status of these lesions did not seem to affect the levels of PD-1+ infiltration (Figure S3F,G).

Taken together, the findings demonstrate a gradual recruitment of immune infiltrates and PD-1/PD-L1 immune checkpoint activation during oral carcinogenesis and suggests that *p53* mutations may alter this dynamic process. The low expression of PD-L1 in carcinomas with *p53* mutations, along with high levels of Tregs and/or decreased levels of CD4+ and CD8+ cells in *p53*-GOF oral lesions, suggest that *p53*-GOF promotes an immune microenvironment that may compromise the efficacy of immune checkpoint inhibitors.

### 3.4. p53^R172H^ Abrogates the Immunopreventive Effects of Anti-PD-1 Antibodies 

**Figure 4:**
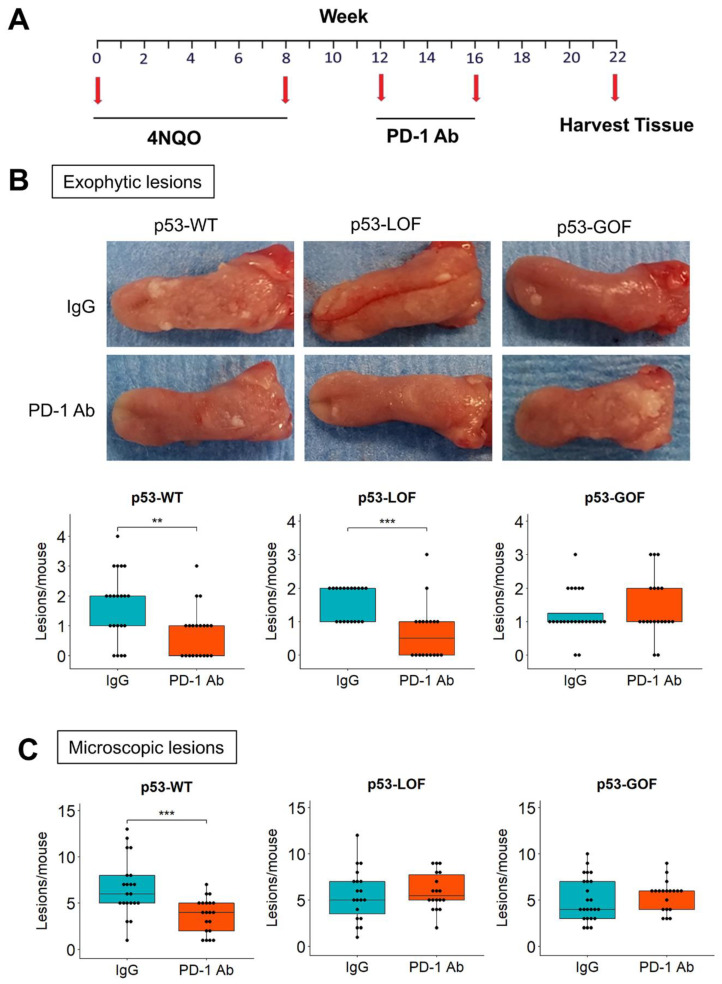
Epithelial mutant *p53^R172H^* blocks the potential of anti-PD-1 to prevent oral tumor development. (**A**) Schedule of the preclinical study for anti-PD-1 oral cancer immunoprevention. (**B**) Gross appearance of the 4NQO-induced exophytic lesions that developed in the tongues of mice treated with control IgG or anti-PD-1. Quantification of the lesions, determined by visual examination, is shown on the bottom panels. (**C**) Quantification of the 4NQO-induced microscopic lesions on histological sections of the tongues. ** *p* < 0.01, *** *p* < 0.001.

To determine whether epithelial *p53* GOF and LOF mutations alter the response to anti-PD-1 during 4NQO-induced oral carcinogenesis, we conducted a preclinical trial with *p53*-GOF, *p53*-LOF, and *p53*-WT mice (Figure 4A). Upon completion of the trial grossly visible exophytic lesions in the tongues of these mice were quantified by visual examination (Figure 4B). We observed a significant decrease in the number of oral tumors that developed in mice that had been treated with anti-PD-1 for both *p53*-WT and *p53*-LOF mice, compared to their respective IgG-treated control mice (Figure 4B). However, the number of tumors that developed in *p53*-GOF mice was similar in control and anti-PD-1 treatment groups. Subsequently, the tongues of these mice were excised and processed for histopathological analyses.

**Figure 5:**
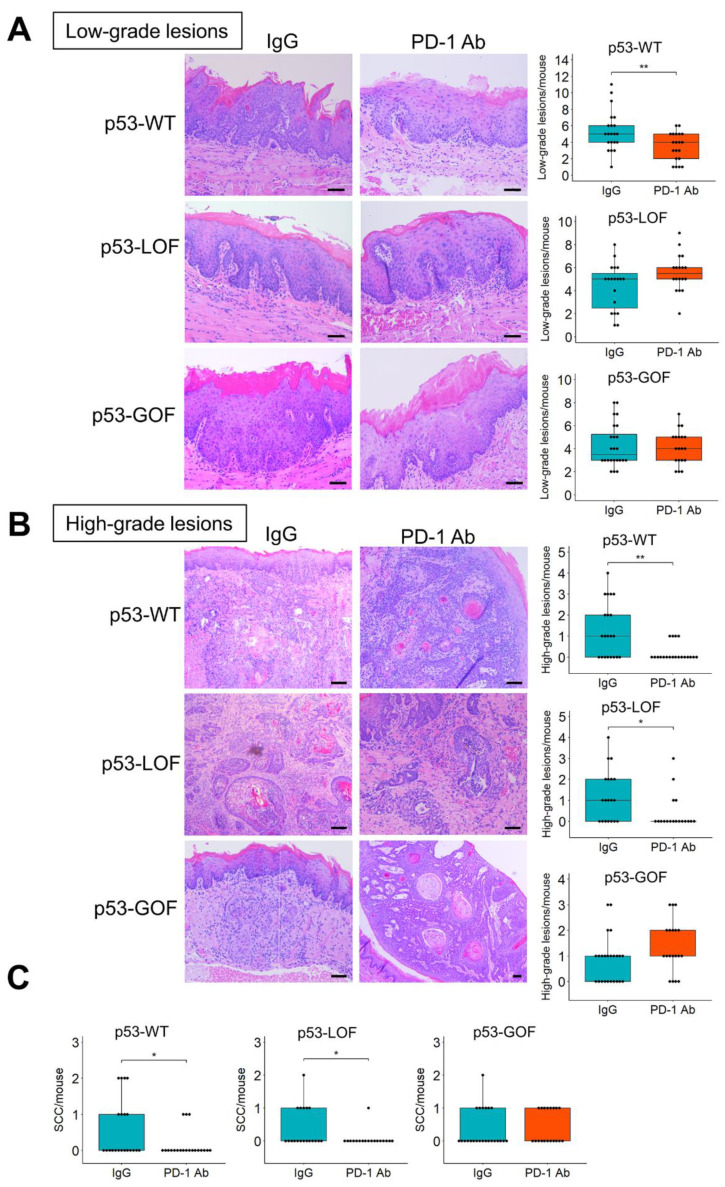
Epithelial mutant *p53^R172H^* blocks the potential of anti-PD-1 to prevent the progression of OPLs. (**A**,**B**) H&E staining of 4NQO-induced (**A**) low-grade lesions or (**B**) high-grade lesions that developed in the tongues of mice treated with control IgG or anti-PD-1. Quantification of the lesions is shown on the right panels. Bar = 100 μm (**C**) Quantification of SCCs in mice treated with anti-PD1 or control IgG. The *p53* status of the mice is indicated at the top of each graph. * *p* < 0.05, ** *p* < 0.01.

Histopathological examination of the oral lesions revealed that the anti-PD-1 treatment in *p53*-WT mice resulted in a significant decrease in the number of microscopic lesions compared to IgG control treatment (Figure 4C). However, in both *p53*-GOF and *p53*-LOF mice, the number of microscopic lesions was similar in control and anti-PD-1 groups. To assess more precisely how *p53* mutations affect OPL development and their progression to SCC, we evaluated the degree of oral dysplasia histopathologically. Our phenotypic evaluation revealed that the anti-PD-1 treatment prevented the development of low-grade lesions in *p53*-WT mice, but not in *p53*-LOF or *p53*-GOF mice (Figure 5A).

Notably remarkable was the significant reduction in the incidence of high-grade lesions in *p53*-WT and *p53*-LOF mice that had been treated with anti-PD-1, compared to their respective IgG controls, indicating that in these mice the anti-PD-1 antibody blocked the progression of OPLs (Figure 5B). However, the number of high-grade lesions was similar in *p53*-GOF mice that had been treated with anti-PD-1 and with control IgG (Figure 5B). Similarly, quantification of only SCCs showed that anti-PD-1 blocked the development of SCCs in *p53*-WT and *p53*-LOF mice, but not in *p53*-GOF mice (Figure 5C). Collectively, these findings demonstrate that epithelial expression of the *p53* GOF mutation *p53^R172H^* in 4NQO-induced oral lesions supports the development of an immune microenvironment that is refractory to reactivation by PD-1 inhibitors and promotes resistance to oral cancer immunoprevention.

### 3.5. Upregulation of IL17 Signaling and Depletion of Exhausted CD8 Cells in the Microenvironment of p53-GOF Papillomas

**Figure 6:**
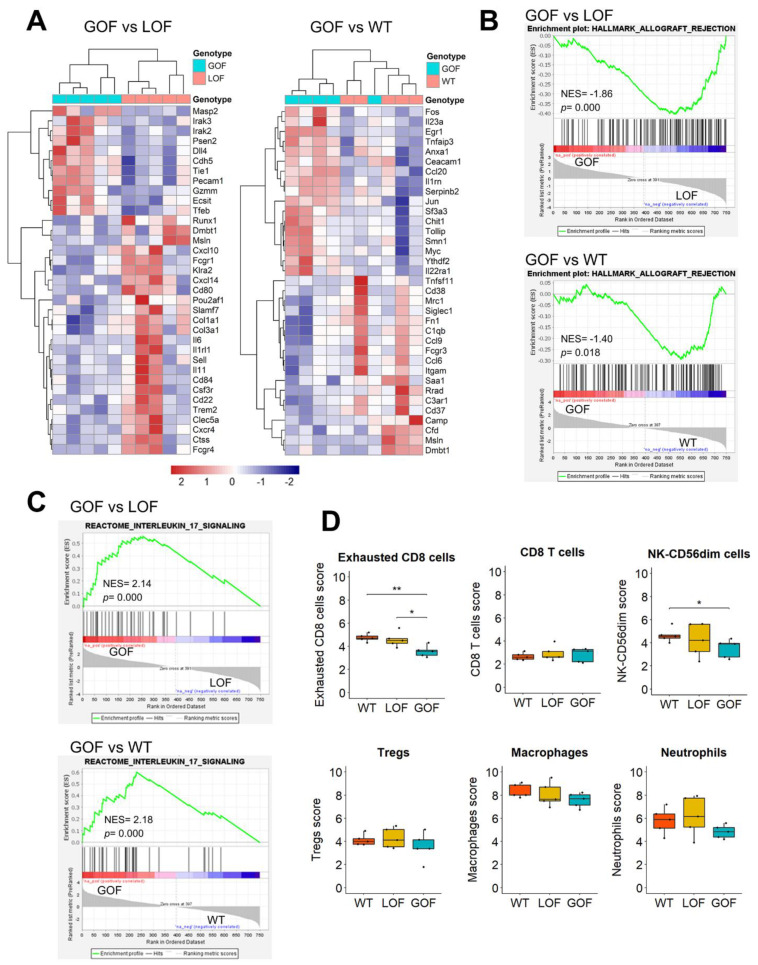
Upregulation of IL17 signaling and depletion of exhausted CD8 cells in *p53^R172H^* papillomas. (**A**) Heatmap of the most differentially expressed genes between 4NQO-induced p53-GOF and p53-LOF papillomas (left panel) and between *p53*-GOF and *p53*-WT papillomas (right panel). (**B**,**C**) Gene Set Enrichment Analysis (GSEA) analysis for the gene sets (**B**) Hallmark Allograft Rejection and (**C**) Interleukin 17 Signaling in *p53*-GOF vs. *p53*-LOF papillomas (top panel) or between *p53*-GOF vs. *p53*-WT papillomas (bottom panel). (**D**) Cell type scores for the indicated immune cell populations determined by nSolver software on *p53*-WT, *p53*-LOF, and *p53*-GOF papillomas. * *p* < 0.05, ** *p* < 0.01.

To gain insights into the mechanisms underlying the resistance to anti-PD-1-mediated oral cancer prevention induced by the *p53* GOF mutation, we analyzed the mRNA expression of 735 immune-related genes using the PanCancer Immune Profiling panel from Nanostring. As our aim was to identify specific immune components of *p53*-GOF OPLs that allows their malignant progression even after treatment with anti-PD-1, we compared the immune profiles of 4NQO-induced *p53*-GOF papillomas with those of *p53*-LOF and *p53*-WT papillomas. This analysis identified 119 genes differentially expressed (*p* < 0.05) between *p53*-GOF and *p53*-LOF papillomas and 100 genes differentially expressed between *p53*-GOF and *p53*-WT papillomas (Figure 6A and Table S5). Interestingly, GSEA analysis revealed that both *p53*-LOF and *p53*-WT papillomas were enriched in genes involved in allograft rejection, compared to *p53*-GOF papillomas, suggesting a weakened cell-mediated immunity in *p53*-GOF lesions (Figure 6B).

To uncover mechanisms of immunosuppression in *p53*-GOF papillomas that may lead to resistance to immunoprevention, we applied GSEA to identify molecular pathways enriched in these lesions. We found that the Interleukin 17 (IL17) signaling pathway was the highest upregulated Reactome pathway in *p53*-GOF papillomas compared to both *p53*-LOF and *p53*-WT papillomas (Figure 6C and Table S6). Although the functional significance of IL17 signaling in oral cancer immunoprevention needs further investigation, recent studies have linked IL17 to resistance to immune checkpoint inhibitors in pancreatic cancer [42].

The PanCancer Immune Profiling platform incorporates a scoring system for a panel of immune cell types, based on the RNA expression of specific sets of markers for each cell type. Notably, we observed that of the 14 immune cell types analyzed, only the exhausted CD8 cell population was found underrepresented in *p53*-GOF papillomas compared to *p53*-LOF and *p53*-WT papillomas (Figure 6D and Figure S4). The NK-CD56^dim^ score was lower in *p53*-GOF papillomas, but only statistically significant compared with *p53*-WT (Figure 6D). Scores for other cell populations, such as macrophages and neutrophils, also showed a trend to be lower in *p53*-GOF lesions, although the differences were not statistically significant (Figure 6D). Since exhausted CD8 cells are a primary target for immune reactivation by PD-1 inhibitors [43], these observations suggest that *p53*-GOF may mediate resistance to immunoprevention of oral cancer, at least in part, through depletion of exhausted CD8 cells in the microenvironment of the oral lesions. 

## 4. Discussion

In this study, we generated mouse models for oral carcinogenesis with genetically engineered epithelial-specific *p53* mutations to determine the effects of *p53* GOF and LOF mutations in oral cancer prevention mediated by PD-1 blockade. Consistent with the oncogenic functions of *p53* GOF mutations, *p53^R172H^* promoted metastasis in OSCCs initiated by the carcinogen 4NQO, and a significantly shorter survival compared to mice carrying *p53*-LOF or *p53*-WT tumors. Remarkably, our results show that PD-1 blockade effectively blocked progression of OPLs in *p53*-WT and *p53*-LOF mice, but not in *p53*-GOF mice. Resistance to PD-1 inhibition was accompanied by upregulation of IL17 signaling and depletion of exhausted CD8 cells in the microenvironment of *p53*-GOF OPLs, which may contribute to the poor response to anti-PD-1. These results indicate that the *p53*-GOF mutation *p53^R172H^* promotes malignant progression of oral tumors and resistance to anti-PD-1-mediated immunoprevention. 

The 4NQO mouse model of oral carcinogenesis faithfully recapitulates the histopathological features observed during human oral cancer development and to display mutational signatures similar to those associated with tobacco smoking, supporting the use of 4NQO as a surrogate for tobacco-induced oral carcinogenesis [30]. By administering 4NQO to mice in which endogenous *p53* GOF or *p53* LOF mutations were activated in oral epithelial cells, we generated mouse models that recapitulate the poor prognosis observed in human head and cancers carrying high-risk *p53* mutations [21,23]. While oral tumors developed faster in both *p53*-GOF and *p53*-LOF mice than in *p53*-WT mice, we found no significant differences between *p53*-GOF and *p53*-LOF mice. However, the *p53* GOF mutation promoted significantly higher incidence of metastasis and shorter survival compared to *p53* LOF or *p53* WT, supporting a role for *p53*-GOF in tobacco-related oral cancer progression and metastasis. This is a significant finding considering that the distinctive effects of *p53* GOF and LOF mutations in oral cancer metastasis could not be determined in previous mouse models where oral tumors where initiated by oncogenic Kras [44]. The newly established mouse models described here provide a platform to perform relevant oral cancer prevention studies and to analyze outcomes in the context of *p53* GOF and LOF mutations. 

Importantly, our delineation of histopathological stages of 4NQO-induced oral lesions allowed for a step-wise assessment of immune infiltrates in progressive stages of oral tumor development. The gradual increase in T-cell infiltration found during oral tumor progression is consistent with simultaneous rise in immunogenic during malignant progression. Interestingly, non-dysplastic oral mucosa from mice that had been exposed to 4NQO showed significantly higher levels of T-cell infiltration than the normal oral mucosa from untreated mice, implying that an immune response can be mounted during the earliest stages of oral tumor development, prior to detection of oral lesions. The progressive recruitment of T-cells during oral tumor progression was accompanied by increased infiltration of immunosuppressive Tregs and PD-1+ cells, consistent with the activation of mechanisms that prevent an effective immune response during oral squamous tumorigenesis. 

In human oral cancers, T cell infiltration has been associated with favorable prognosis [45,46], but the clinical implications of Treg infiltration remains uncertain since it has been found associated with poor survival [47], and also with better outcomes in OSCC [48,49]. The impact of infiltrating Tregs on clinical outcomes may depend on tumor stage, suggesting that other elements of the tumor microenvironment could modulate their function in oral tumors [47]. The increased Treg infiltration observed in *p53*-GOF early lesions compared to *p53*-LOF and *p53*-WT lesions, and the significant accumulation of Treg infiltration during the transition of *p53*-GOF papillomas to carcinomas suggest that Treg recruitment could be regulated by *p53* GOF mutations. Further investigations are needed to determine the clinical significance of Treg infiltration in oral tumor development, in the context of *p53* mutations. 

Our findings are consistent with recruitment of PD-1+ T-cells and induction of PD-L1 in epithelial cells of the oral lesions in early stages of oral carcinogenesis. Although PD1 and PD-L1 tended to increase during progression from papilloma to SCC, the differences were not statistically significant. This could be at least partially due to the low levels of PD-L1 found on *p53*-GOF and LOF SCCs compared to *p53*-WT SCCs. PD-L1 expression has been shown to correlate with favorable prognosis in several human solid malignancies, including head and neck cancer [50,51], consistent with the better prognosis of *p53* WT human head and neck cancers, and with the longer survival rates of *p53* WT mice compared to mice with *p53* mutations found in this study.

The T-cell infiltration and recruitment of immunosuppressive Tregs and PD-1+ cells during the early stages of oral carcinogenesis suggest that blocking immunosuppressive activities in the microenvironment of OPLs may stimulate an anti-tumor immune response. Moreover, the dynamics of immune cell infiltration in oral lesions that differ in their *p53* status suggested that the response to immune checkpoint blockade may vary depending on the *p53* content. Our preclinical studies support this hypothesis since anti-PD-1 was very effective in preventing the progression of *p53*-LOF and *p53*-WT OPLs. In contrast, activation of the *p53^R172H^* mutation in oral epithelial cells induced resistance to anti-PD-1-mediated immunoprevention. These findings underscore potential clinical implications for OPLs with *p53* GOF mutations which may not respond to PD-1 blockade and suggest that *p53* status may be a relevant molecular biomarker to predict response in oral cancer immunoprevention.

Our immune expression profiling uncovered potential mechanisms that may confer *p53*-GOF papillomas with resistance to PD-1 inhibitors. IL17 signaling was the leading pathway found to be upregulated in *p53*-GOF papillomas. This finding could be relevant for oral cancer immunoprevention, as IL17 signaling is emerging as a potent immunosuppressive mechanism that promotes resistance to immune checkpoint blockade, although the mechanisms underlying these effects are still poorly understood [42]. Our data suggest that *p53*-GOF mutations could promote an immunosuppressive microenvironment by upregulating IL17 signaling. Interestingly, we found depletion of exhausted CD8 cells in *p53*-GOF papillomas compared to *p53*-LOF and *p53*-WT papillomas. Exhausted CD8 cells lose their T-cell function over time as a result of persistent antigen exposure [52]. Although these cells are eventually depleted from the tumors, they can be reactivated with immune checkpoint inhibitors [43]. Since the exhausted CD8 cell population is significantly depleted in *p53*-GOF papillomas, these tumors are expected to be more refractory to anti-PD-1, as we observed in our studies. Our results highlight the contribution of epithelial *p53*-GOF mutation to induce an immunosuppressive tumor microenvironment that lacks immune checkpoint target cells. Further investigation will be needed to dissect the interplay between *p53* mutations and IL17 signaling and the potential consequences for T cell exhaustion in oral cancer development and in response to PD-1 blockade.

## 5. Conclusions

This study demonstrates that activation of an endogenous *p53* GOF mutation in epithelial cells of carcinogen-induced oral tumors promotes resistance to anti-PD-1-mediated immunoprevention, associated with upregulation of IL17 signaling, and lack of exhausted CD8 T cells in the OPL microenvironment. These findings support the use of *p53* profiling to predict the preventive effects of PD-1 inhibitors to control the progression of OPLs.

## Data Availability

The data presented in this study are available upon request from the corresponding author.

## Supplementary Materials

The following are available online at https://www.mdpi.com/2072-6694/13/6/1471/s1. Figure S1: Mouse genetic background. Figure S2: Genomic analysis of p53. Figure S3: Quantification of the immunohistochemistry. Figure S4: Cell type scores. Table S1: Number of samples analyzed by immunohistochemistry. Table S2: Nanostring counts. Table S3: Primers used for p53 sequencing. Table S4: Tumor kinetics and metastasis rates. Table S5: Immune Profiling. Table S6: Reactome GSEA analysis.
