# Supplementary material for: Epithelial Mutant p53 Promotes Resistance to Anti-PD-1-Mediated Oral Cancer Immunoprevention in Carcinogen-Induced Mouse Models
Source: Cancers (Basel). 2021 Mar 23;13(6):1471. doi: 10.3390/cancers13061471 (PMC8005156; doi:10.3390/cancers13061471)
Supplement: Supplementary file 1 [file cancers-13-01471-s001.zip › Supplementary Figures S1-S4.pdf]

Supplementary Figure S1

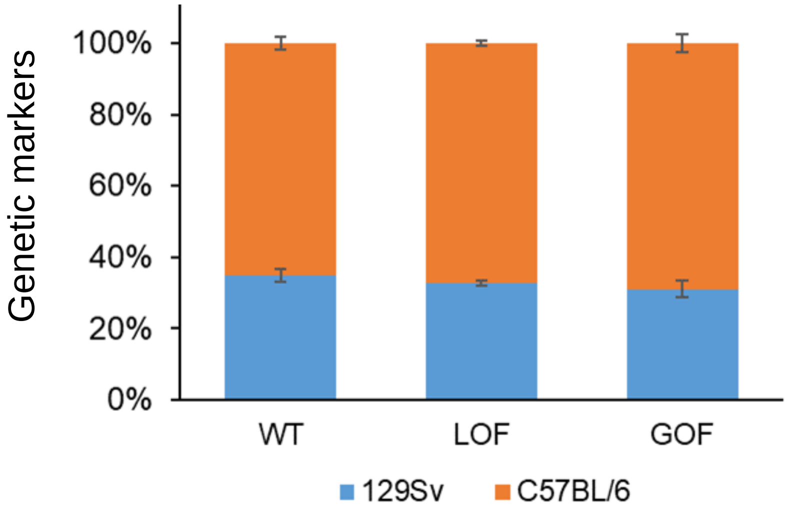

Supplementary Figure S1. Genetic background of mice used in this study. The percentage of specific genetic markers for 129Sv and C57BL6 strains is represented for p53-WT mice (n=6), p53-LOF mice (n=3) and p53-GOF mice (n=3). Note that the genetic background is similar for all groups. Comparative analyses for differences between each group against each of the other two groups were not statistically significant ($p>0.05$).

Supplementary Figure S2

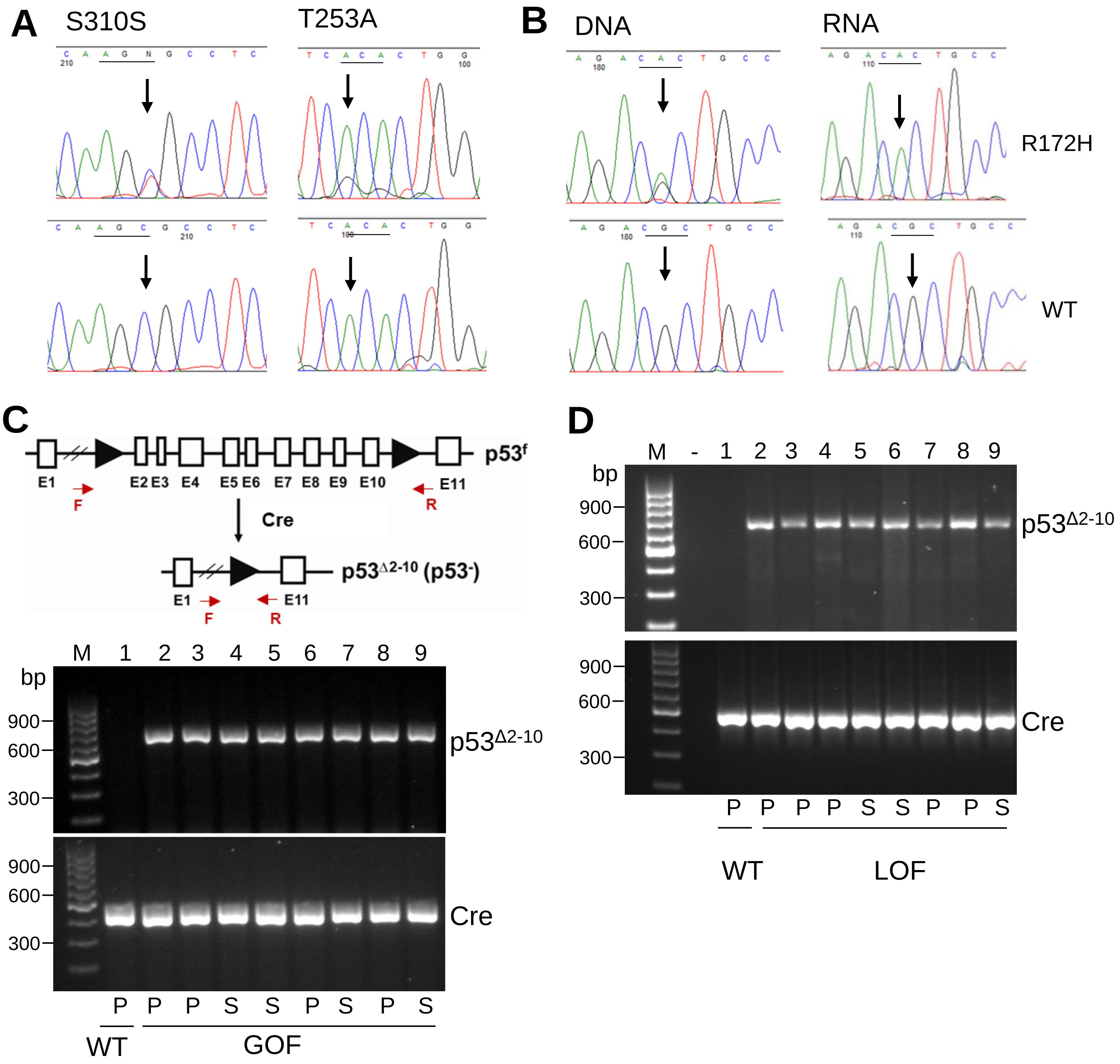

Supplementary Figure S2. Genomic analysis of p53 in papillomas and SCCs induced by 4NQO. (A) Sequencing profiles of the AGC to AGT mutation encoding for S310S, and ACA to GCA encoding for T253A found in p53-WT SCCs. Top panel represents the mutant tumor and bottom panels represent wild type tumors. (B) The CAC codon encoding for R172H was detected in all p53-GOF tumors (top panels), but not in p53-WT tumors (bottom panels), and expression of p53-R172H was confirmed by RNA sequencing (right panels). (C) Activation of the $p53^{\Delta 2\text{-}10}$ deletion in the remaining p53 allele in p53-GOF tumors. The top panel shows an schematic representation of the floxed p53 allele ($p53^{f}$) and position of the primers used to detect deletion of the loxP-flanked p53 sequences. Bottom panels show PCR identification of the $p53^{\Delta 2\text{-}10}$ deletion in GOF tumors (P: papillomas; S: SCCs). A WT tumor (lane 1) was used as negative control. Cre amplification was used as positive control for DNA integrity and PCR conditions. (D) Activation of the $p53^{\Delta 2\text{-}10}$ deletion in p53-LOF tumors.

Supplementary Figure S3

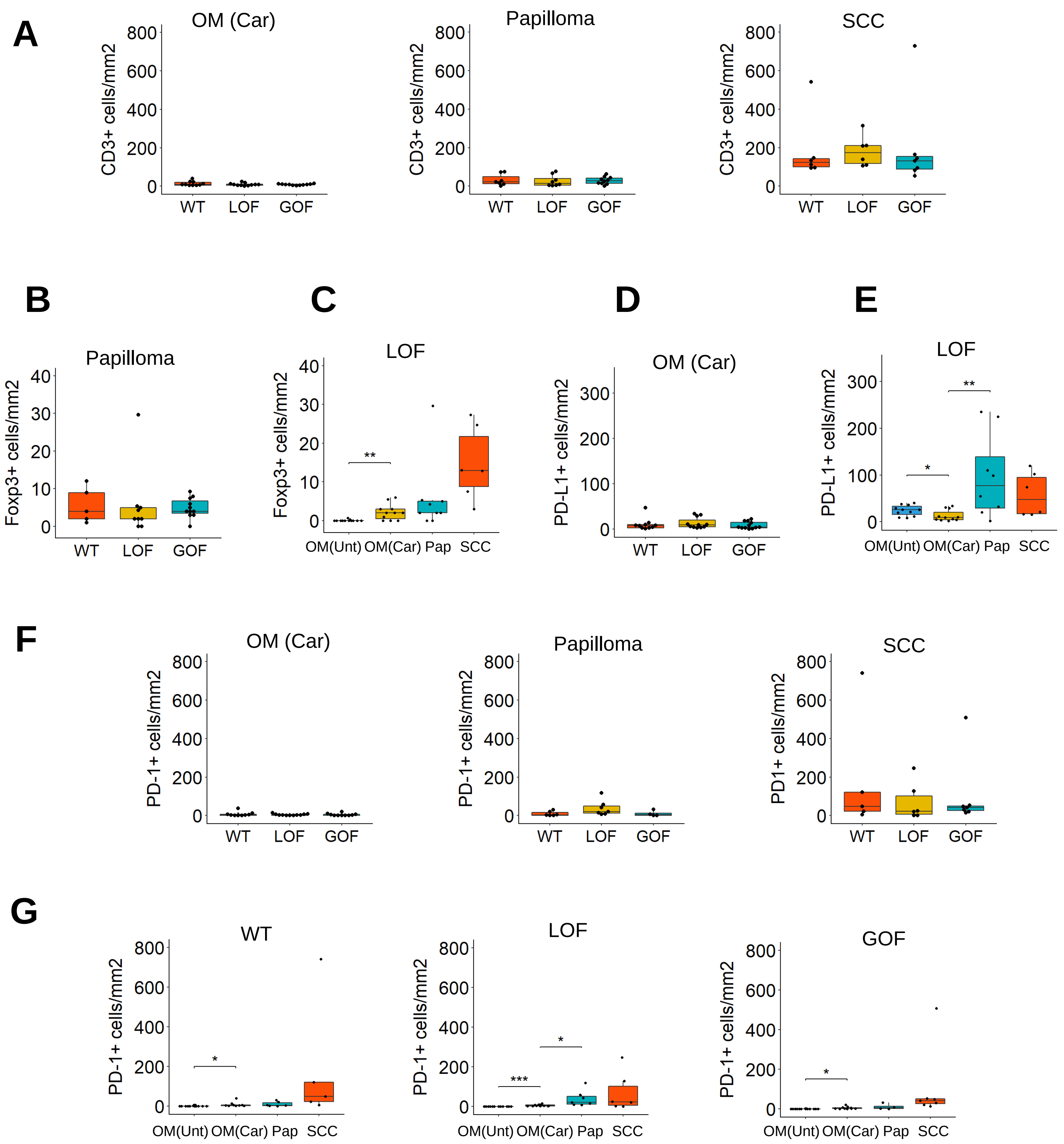

Supplementary Figure S3. (A) Quantification of the immunohistochemistry shown in Figure 2 for CD3. (B-G) Quantification of the immunohistochemistry shown in Figure 3 for (B-C) Foxp3, (D-E) PD-L1 and (F-G) PD-1. *p<0.05, **p<0.01, ***p<0.001.

Supplementary Figure S4

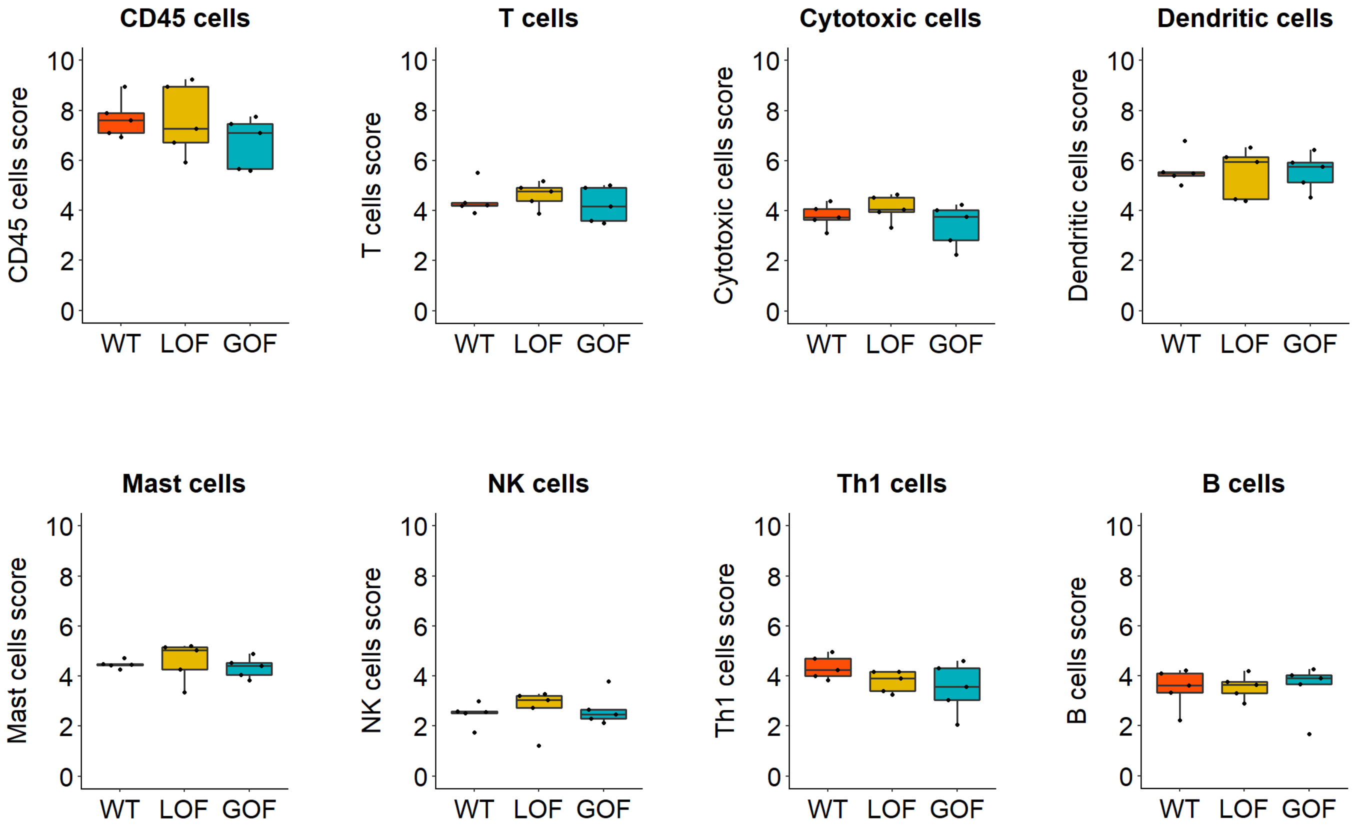

Supplementary Figure S4. Cell type scores for each of the indicated cell populations determined by nSolver software on papillomas that developed in *p53*-WT, *p53*-LOF and *p53*-GOF mice.
